# SARS-CoV-2 BA.2 (Omicron) variant infection in pediatric liver transplanted recipients and cohabitants during 2022 Shanghai outbreak: a prospective cohort

**DOI:** 10.1186/s12985-023-01978-4

**Published:** 2023-02-11

**Authors:** Xin-ye Zhu, Ye-feng Lu, Feng Xue, Yi Luo, Ming-xuan Feng, Bi-jun Qiu, Tao Zhou, Jian-jun Zhu, Jian-jun Zhang, Ping Wan, Qiang Xia

**Affiliations:** 1 Department of Liver Surgery, Ren Ji Hospital, Shanghai Jiao Tong University School of Medicine, 1630 Dongfang Road, Shanghai, 200127 China; 2 Shanghai Institute of Organ Transplantation, 1630 Dongfang Road, Shanghai, 200127 China; 3 Shanghai Research Center of Organ Transplantation & Immune Engineering Technology, 1630 Dongfang Road, Shanghai, 200127 China

**Keywords:** Children, Immunosuppression, Liver transplantation, SARS‐CoV‐2, Solid organ

## Abstract

**Background:**

The Omicron variant BA.2 was the dominant variant in the COVID-19 outbreak in Shanghai since March 2022. We aim to investigate the characteristics of SARS-CoV-2 Omicron variant infection in pediatric liver-transplanted recipients.

**Methods:**

We conducted a single-center, prospective, observational, single-arm study. We enrolled pediatric liver-transplanted patients infected with the Omicron variant BA.2 from March 19th to October 1st, 2022 and analyzed their demographic, clinical, laboratory, and outcome data. The management of COVID-19 was conducted according to the 9th trial edition of the Chinese guideline. The immunosuppressive therapy was tailored considering the patients’ infection developments and liver functions.

**Results:**

Five children were included. The primary diseases included Niemann-Pick disease, propionic acidemia, decompensated cirrhosis, biliary atresia, and Crigler-Najjar syndrome type I. All of the patients were onset with fever before or when getting RNA-positive results at the age of 3 (Range: 1–13) years. The infection duration was 29 (Range: 18–40) days. Three and two children were diagnosed with mild and moderate COVID-19 respectively. Two patients were tested RNA-positive within 14 days after having been tested negative. The immunosuppressants were paused or extenuated in four patients. Eight of all nine cohabitants were injected with at least two doses of inactivated SARS-CoV-2 vaccine. The disease courses were significantly longer than the patients (*P* < 0.05).

**Conclusions:**

Post-transplant immunosuppression slows down the virus clearance and increases the risk of relapse but does not affect symptom duration or infection severity in pediatric patients. Patients can usually gain a favorable outcome and prognosis by extenuating immunosuppressants.

## Background

Severe Acute Respiratory Syndrome Coronavirus 2 (SARS-CoV-2) variant B.1.1.529 (omicron), first seen in South Africa, was designated by WHO as a new variant of concern on November 26^th^, 2021 [1]. Since then, omicron spread to over 80 countries [2]. The Omicron variant BA.2 showed superspreading potential during the outbreak in Hongkong since January, 2022 [3, 4] and then became the predominant variant which caused the Corona Virus Disease 2019 (COVID-19) outbreak in Shanghai beginning in the second week of March 2022 [5]. Studies have already discovered higher transmissibility and less severity than the delta variant and the wild type [6–9].

Immunosuppression treatment is essential to liver transplant (LT) recipients to avoid allograft rejection, while an immunosuppressive state raises the risk of respiratory infection of various kinds of the pathogen [10, 11]. Liver-transplanted patients have an increased risk of acquiring COVID-19 and a longer infection duration [12]. In the earlier period of COVID-19, liver injury was associated with higher mortality and intensive care unit admission in infected LT recipients [13, 14]. Also, the liver is a direct target organ of SARS-CoV-2 infection which elevates liver enzymes and causes histologic changes in the liver [15]. What makes things worse is that the safety and effectiveness of SARS-CoV-2 vaccination in post-LT recipients is still controversary [16–18]. Hopefully, the mortality rates of chronically immunosuppressed LT recipients are lower than the general population [12]. Current studies indicated that COVID-19 did not deteriorate the general status as well as the allograft liver in immunosuppressed post-LT children [19]. It is to be clarified how the immunosuppressive state interacts with the development of COVID-19.

Several studies have investigated the LT recipients infected with the wild type or the Delta variant [19–30]. Yet there were no available data in LT recipients infected with the Omicron variant, which is very important considering the complex mutation of Omicron variants in pathogenicity and immunogenicity. Here, we report the clinical presentation, laboratory results, treatment and prognosis of 5 liver transplanted recipients infected with the Omicron variant during the 2022 Shanghai Outbreak and those of their cohabitants.

## Methods

### Study design

This is a single-center, non-controlled, prospective, observative clinical trial conducted at the Department of Liver Surgery, Renji Hospital, School of Medicine, Shanghai Jiao Tong University. Participants were enrolled from March 19th, 2022 to October 1st, 2022. The follow-up for this study was stopped on December 1st, 2022.

### Study population and patient selection

We included all children and adolescents (aged ≤ 18 years) who had accepted liver transplantation in our center and had been diagnosed as COVID-19 in Shanghai according to the criteria in the Scheme for Diagnosis and Treatment of 2019 Novel Coronavirus Pneumonia (The 9th Trial Edition), hereinafter referred as “The 9th Trial Edition” published on March 16th [31]. SARS-CoV-2 infections were diagnosed by a real-time reverse transcriptase polymerase chain reaction assay (RT-qPCR) of nasal and pharyngeal swab specimens. To exclude heterotopic infections outside Shanghai, we reviewed the patients’ and cohabitants’ itinerary records and RT-qPCR records within 2 weeks before the COVID-19 diagnosis, which were routinely tested in the community according to the local risk level.

### Pre-treatment preparation

Before the initiation of therapy, the following data were documented for all patients: (1) baseline demographic characteristics, including gender, age, height, and weight; (2) LT operation records and post-LT follow-up records, including LT complications and corresponding treatments; (3) detailed medical history, including epidemiologic characteristics, symptoms, and vaccine injection history—symptoms concerned included fever (duration, max, max date, fever type), respiratory symptoms (dry cough, nasal congestion, chest pain, breathing difficulty, sore throat, etc.), loss of smell and taste, fatigue, anorexia, nausea, diarrhea, and myalgia [2]; (4) other evidence of SARS-CoV-II infection, including gene sequencing from the patient’s specimen, serum specific antibodies, viral load and CT scan; (5) clinical examination including comprehensive blood panel, involving serum C‐reactive protein (CRP), blood routine, serum creatine, alanine aminotransferase (ALT), aspartate aminotransferase (AST), total protein, albumin, alkaline phosphatase (ALP), gamma‐glutamyl transferase (γ-GT), direct bilirubin (DB). If necessary, examination would be done to record myocardial injury (brain natriuretic peptide, troponin), coagulation function (APTT, PT, INR, D-dimmer, fibrinogen), and immune function (IL-6, T cell subsets).

### Treatments and outcomes of SARS-CoV-2 infection

Treatments were applied, if necessary, according to “The 9th Trial Edition,” including fluid management, oxygen therapy, antipyretic therapy, antibiotic therapy, anticoagulant therapy, posture therapy and mental interference. The start of the infection course was defined as the onset of typical symptoms or otherwise the first day of positive RT-qPCR results. The end of the infection course was defined as the first day of two consecutive times of negative RT-qPCR results (the limit value of cycle threshold value of *N* and *ORF* of is 40; the interval should be at least 24 h) including relapse. After being cured, the infected patients and cohabitants were classified as asymptomatic, mild, moderate, severe, and critical according to the clinical presentation, laboratory examination, and chest radiography findings. Asymptomatic: cases without any clinical and radiological findings. Mild: cases with upper respiratory tract symptoms and normal respiratory system examination. Moderate: cases with abnormal radiological findings of pneumonia without the symptoms of dyspnea and hypoxemia. Severe: cases with high fever for over three days; or cases with polypnea unaccompanied with fever and crying; or cases with arterial oxygen saturation of < 93%; or cases with auxiliary breathing such as flaring of alaenasi or three concave signs; or cases with convulsion and lethargy; or cases with sitieirgia or feeding intolerance accompanied with exsiccosis. Critical: cases who develop respiratory failure and need mechanical ventilation; or cases who develop shock; or cases with other organ failures who need intensive care unit. The occurrence of COVID-19 sequelae was evaluated in the 2nd month after discharge according to symptoms and RT-qPCR results [32]. To reduce the possibility of an association with possible previous infection, reinfection is defined as a positive RT-qPCR result at least 90 days after the end of the latest infection course.

### Post-LT Immunosuppression

The initial immunosuppressive therapy consisted of tacrolimus (TAC) and prednisone acetate (PA). Steroids were weaned off within 3 months post‐transplant. Mycophenolate mofetil (MMF) was introduced when the tacrolimus concentration in blood had not reached the goal even by increasing the dose. Cyclosporin A (CsA)-based immunosuppressive therapy replaced the TAC-based therapy if MMF had not helped or there had been indispensable adverse effects. Blood routine, liver function, and immunosuppressant plasma concentration were uniformly done in out-patient department and recorded as evidence of immunosuppressant adaption. The post-LT follow-up was routinely done at a weekly frequency in the first three months, bi-weekly in the first half-year, and monthly in the first year.

### Statistical methods

We reviewed the medical files of all the patients to collect their epidemiologic, demographic, clinical, laboratory, and outcome data by using a standardized study specific form. We described patients’ characteristics with medians, ranges and percentages. SPSS software was used for further statistical analysis. Kolmogorov–Smirnov test was used to check normality. For data conforming to a normal distribution, paired t-test was used to assume a two-tail hypothesis with *P* < 0.05. Non-parametric tests were used to test data that do not conform to a normal distribution.

## Results

### Patients’ characteristics

At the start of the Shanghai outbreak dominated by Omicron variant BA.2, 2805 pediatric recipients were under regular follow-up. Five post-LT patients, as well as nine cohabitants, were included in our study. All of them had been staying in Shanghai since the month before the outbreak and none of them had been in Wuhan or foreign countries, so none was excluded due to heterotopic infections. The baseline characteristics of those 5 patients are detailed in Table 1. There were 3 boys and 2 girls. The median age of the patients in diagnosis were 3 (Range: 1.17–13) years old. The median height was 92 (Range: 78–148) cm, and the median weight was 19 (Range: 8.5–61) kg. The primary disease included Niemann-Pick disease, propionic acidemia, decompensated cirrhosis, biliary atresia, and Crigler-Najjar syndrome type I. The donor type included whole liver transplantation (WLT; n = 2), living donor liver transplantation (LDLT; n = 2), and split liver transplantation (SLT; n = 1). The median of graft volume/recipient body weight ratio (GRWR) was 3.73% ranging from 2.16 to 3.73%. No patient developed surgical complications. As for non-surgical complications, one patient developed post-transplant lymphoproliferative disorders (PTLD), another developed de novo HBV infection and tuberculosis, and the others developed no post-LT complication. The median interval between LT and COVID‐19 infection was 99 days, ranging from 27 to 851.

**Table 1:** Patients’ Characteristics

	Gender	Age	Height (cm)	Weight (kg)	LT indication	Operation Date	Donor type	GRWR (%)	Post-LT Complication	Interval from LT to COVID-19 infection (day)
Case A	Female	10y	120	61	Niemann-Pick disease	2/4/2022	WLT	2.19	None	59
Case B	Female	1y 2mon	78	8.5	Propionic acidemia	1/21/2020	LDLT	3.73	None	99
Case C	Male	13y	148	34	Decompensated cirrhosis	12/28/2021	WLT (retransplantation)	2.16	None	27
Case D	Male	2y 7mon	92	19	Biliary atresia	3/12/2022	SLT	3.56	PTLD	739
Case E	Male	3y	90	15	Crigler-Najjar symdrome type I	4/6/2020	LDLT	3.80	de novo HBV infection, Tuberculosis	851

*GRWR* graft volume/recipient body weight ratio, *LT* liver transplantation, *WLT* whole liver transplantation, *LDLT* living donor liver transplantation, *SLT* split liver transplantation, *PTLD* post‐transplant lymphoproliferative disease, *HBV* Hepatitis B Virus

### Disease course

**Fig. 1:**
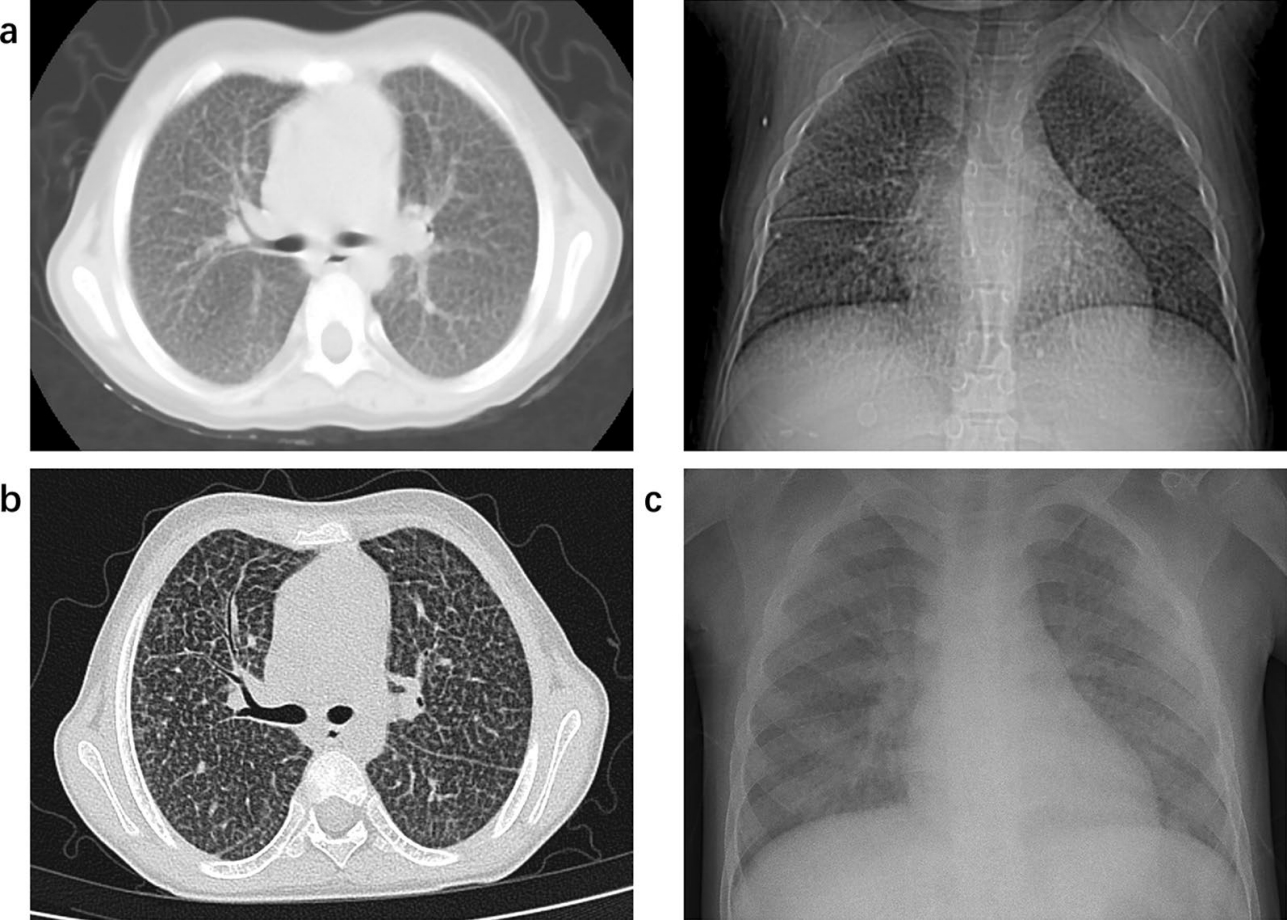
The chest radiography of Patient A. (**a**) post-LT: typical imaging presentation of Niemann-Pick Disease; (**b**) in the early stage (the 2nd day) of SARS-CoV-2 infection: no visible change; (**c**) in the later stage (the 21st day) of SARS-CoV-2 infection: increased and blurred texture in both lungs with patchy exudate

The most common onset symptom was fever (n = 4, 80%) (Table 2). The median patient maximum body temperature was 38.5 (Range: 38–39.9) °C for a median duration of 2 (Range: 2–5) days. In contrast, fever happened to less than 40% of patients in adult studies [2]. Other symptoms included anorexia (n = 2), fatigue (n = 1), dry cough (n = 1), sputum sounds (n = 1), fatigue (n = 1), monophagia (n = 1), and myalgia (n = 1). Upper respiratory symptoms, such as running nose, sore throat, sneezing, or nausea, were not seen in our patients, which occurred on over 50% of patients in the elder age group [2]. None of the patients accepted the SARS-CoV-2 vaccine. Four (80%) patients got positive results in RT-qPCR after symptoms. The interval from the onset of symptoms to a positive result in RT-qPCR was 2 (Range: 0–6) days. The median length of infection was 29 (Range: 19–40) days. Three children were diagnosed with mild COVID-19 while two others were diagnosed moderate; none were diagnosed asymptomatic, severe, or critical. The positive result of RT-qPCR was detected for a median of 15 days, ranging from 13 to 23 days. Patient D was co-infected with Epstein–Barr virus (EBV) and Patient E with hepatitis B virus (HBV) and mycobacterium tuberculosis (TB). The other three patients were not infected with other pathogens. Pneumonia manifestation on chest radiography was seen in Patient A on the 21^st^ day of infection (Fig. 1). Most patients experienced a slight increase in alanine transaminase (ALT) and aspartate transaminase (AST) during the infection.

**Table 2:** Patients' Disease Courses of COVID-19 infection

		Case A	Case B	Case C	Case D	Case E
Presentation		Fever for 2 days, max 39.9 °C	Fever for 5 days, max 38.3 °C, with fatigue and anorexia	Fever for 2 days, max 38.5 °C, with sputum sounds	Fever for 2 days, max 39.8 °C, with dry cough, fatigue, monophagia, and myalgia	Fever for 2 days, max 38 °C
Injected doses of COVID-19 vaccine		0	0	0	0	0
Interval from symptom onset to RT-qPCR positive (day)		1	6	0	2	2
Length of the infection course (day)		40	29	39	19	18
Severance of COVID-19†		Mild	Mild	Moderate	Mild	Moderate
RT-qPCR positive duration (days)		23	13	13	17	15
Co-infection		None	None	None	EBV	HBV, TB
Chest radiography		Pneumonia	-§	-	Normal	multiple calcification
Blood routine‡	WBC *3.5–9.5* × *10^9/L*	4.86	-	1.23	28.39↑	10.26↑
	Lymphocytes (#) *1.2–3.4* × *10^9/L*	1.61	-	0.19	2.9	5.7↑
	CRP‐max *0–8 mg/L*	9.63	-	16.26↑	3.7	40.78↑
Liver function‡	ALT *9–50 IU/L*	16	-	56↑	63↑	56↑
	AST *15–40 U/L*	63↑	-	57	84↑	57
	γ-GT *10–60 U/L*	145↑	-	42	30	42
FK/CSC C0 level		197.40	-	7.30	2.30	52.00

†The mild is defined as cases with upper respiratory tract symptoms and normal respiratory system examination. The moderate is defined as cases with abnormal radiological findings as pneumonia without the symptoms of dyspnea and hypoxemia

‡The blood routine and liver function were tested at the first diagnosis of COVID-19

§”-”in this table means that the patient didn’t accept the examination

*max* maximum body temperature, *COVID-19* Corona Virus Disease 2019, *RT-qPCR* real-time reverse transcriptase polymerase chain reaction assay, *HBV* Hepatitis B Virus, *EBV* Epstein–Barr Virus, *TB* Mycobacterium Tuberculosis, *WBC* white white blood cell, *CRP* C‐reactive protein, *ALT* alanine transaminase, *AST* aspartate transaminase, *γ-GT* gamma‐glutamyl transferase, *FK/CSC C0 level* the initial trough concentration of FK 506 or cyclosporin

### Treatment and outcome

Ibuprofen was applied to Patient B, Patient C, and Patient D to control the fever in the very early period of COVID-19 (Table 3). All the patients except Patient A accepted antibiotic drugs to handle suspected bacterial co-infection. During the infection, three patients extenuated the doses of the immunosuppressants, and two removed the immunosuppressants. At discharge, three of the five patients took lower dosage of immunosuppressants than before the SARS-CoV-2 infection. Most patients were hospitalized once and the hospitalization lasted for a median of 21 (Range: 0–46) days (Table 3). All the patients were cured and alive. The median follow‐up after the diagnosis of COVID‐19 was 203 (Range: 140–228) days. In the latest follow-up, the results of laborator examination were mostly in the normal range. Till the latest follow-up, none of them had developed acute or post-acute COVID-19 sequelae in pulmonary, cardiovascular, hematological, diabetic, gastrointestinal, renal, mental health, musculoskeletal or neurological system [33].

**Table 3:** The treatments and outcomes of patients

		Case A	Case B	Case C	Case D	Case E
Treatment for COVID‐19	Antipyretic	None	Ibuprofen	Ibuprofen	Ibuprofen	None
	Antibiotic	None	Meropenem	Cefdinir	Meropenem	Cefixime → Cefoperazone and Sulbactam
Immunosuppression treatment	Pre-COVID‐19	CsA 80 mg bid + PA 7.5 mg qd + MMF 0.5 g bid	CsA 40 mg bid + MMF 0.25 mg qd	TAC 2.5 mg bid	TAC 2.5 mg bid	CsA 0.15 mg
	During COVID‐19	Extenuate MMF first, then PA, finally to CsA 45 mg bid	CsA 30 mg bid	Extenuate TAC three times to 1.0 mg-1.5 mg	Stop all the immunosuppressant	Stop all the immunosuppressant
	At discharge	CsA 45 mg bid	CsA 30 mg bid + MMF 0.25 mg qd	TAC 1.0 mg-1.5 mg bid	TAC 2.5 mg bid	none
	At the latest follow-up	CsA 60 mg bid	CsA 30 mg bid	TAC 2.0 mg-1.5 mg	TAC 2.5 mg bid	CsA 0.15 mg
Hospitalization	Times	2	1	1	1	1
	Total duration (day)	46	0	21	27	19
COVID-19 outcome		Cured	Cured	Cured	Cured	Cured
Follow‐up duration after the diagnosis of SARS-CoV-2 infection (day)		213	228	203	140	167
COVID-19 sequelae or reinfection		None	None	None	None	None
Blood test at the latest follow-up	WBC*3.5–9.5* × *10^9/L*	11.77↑	8.9	2.4	10.91	9.73
	Lymphocytes (#)*1.2–3.4* × *10^9/L*	4.29↑	3.52↑	25.1	1.64	4.16↑
	CRP*0-8 mg/L*	< 0.5	< 0.5	< 0.5	< 0.5	< 0.5
	ALT*9-50 IU/L*	31	46	24	16	40
	AST*15-40 U/L*	44↑	64	22	38	67
	γ-GT*10-60 U/L*	42	16	19	9	16
Newly-onset LT complication		None	None	None	None	None

*COVID-19* Corona Virus Disease 2019, *SARS‐CoV‐2* Severe Acute Respiratory Syndrome Coronavirus 2, *TAC* – tacrolimus, *PA* prednisone acetate, *MMF* mycophenolate mofetil, *CsA* cyclosporin A, *WBC* white white blood cell, *CRP* C‐reactive protein, *ALT* alanine transaminase, *AST* aspartate transaminase, γ-GT gamma‐glutamyl transferase

**Fig. 2:**
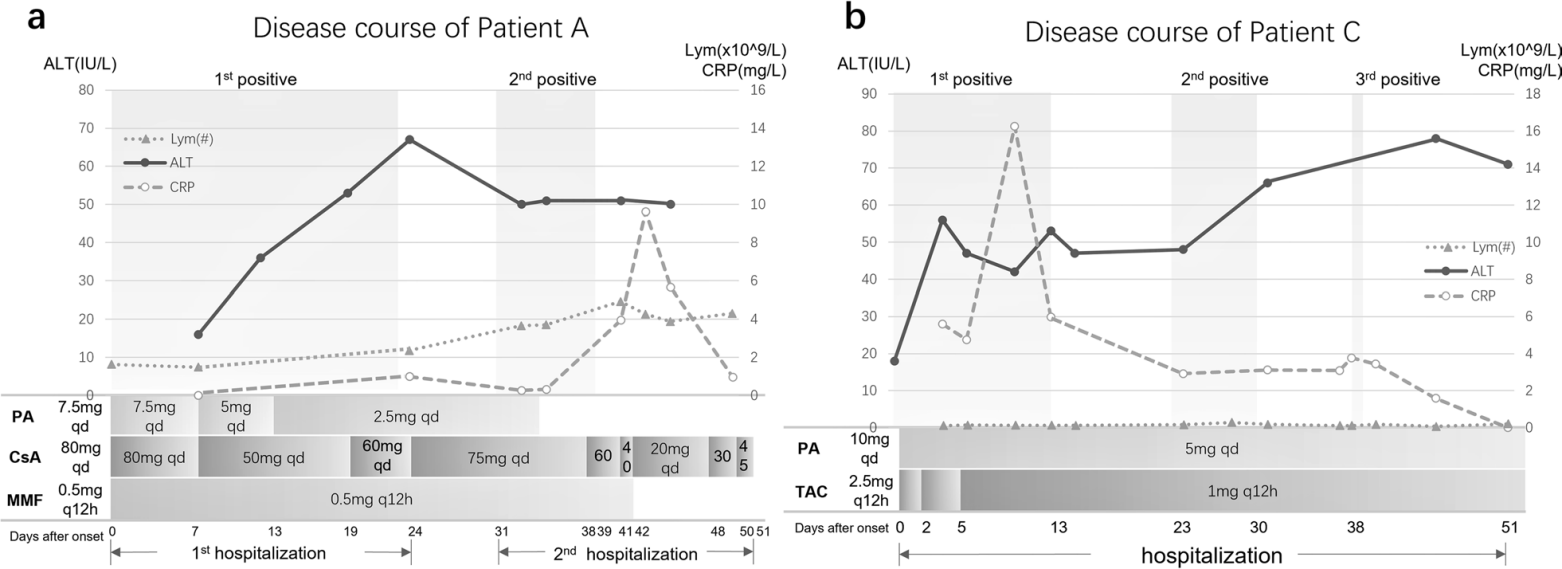
The disease course of the two relapse patients. (**a**) Patient A; (**b**) Patient C. *lym(#)* lymphocyte count, *ALT* alanine transaminase, *CRP* C‐reactive protein, *TAC* tacrolimus, *PA* prednisone acetate, *CsA* cyclosporin A, *MMF* mycophenolate mofetil, *q12h* every 12 h, *qd* every day

Without any other clinical presentation, two patients were tested positive again for RT-qPCR within 14 days after having been tested negative. The positive result duration became shorter with the increase in recurrence (Fig. 2). After an 8-day interval, Patient A was again tested positive in RT-qPCR without any symptoms, which caused her second hospitalization (Fig. 2a). Though the RT-qPCR result returned negative within 8 days, her ALT stabilized at a slightly high level of about 50 IU/L. Patient A’s CRP arose after the earlier infection and reached its peak at 9.63 mg/L, seemingly associated with the dosage of the immunosuppression therapy. Her lymphocyte count was gently increasing and remained high in the range from 4.99 to 6.64 × 10^9^/L. After discharge, Patient A’s liver function results and CRP respectively returned normal on Day 63 and Day 42. The dose of CsA was raised to 60 mg bid on Day 56. Her lymphocyte count had not yet returned to the reference value. As for Patient C, his lymphocyte count was continuously low, which had begun before the COVID-19 infection (Fig. 2b). His TAC was extenuated from 2.5 mg every 12 h (q12h) to 1.0 mg q12h in the first 5 days and maintained till the latest follow-up. Immunoglobin G was injected at 10 g every day (qd) for 3 courses during the first positive period. On Day 10, his CRP reached its peak at 16.26 mg/L. Cefdinir was applied on Day 11 and the CRP was controlled on Day 13, which had been decreasing since then and was corrected on the same day. Patient C’s second positive result in RT-qPCR occurred at an interval of 10 days and lasted for 7 days (Fig. 2b). His ALT continued rising and reached 78 UI/L. He had not left hospital until his COVID-19, evaluated by CRP and RT-qPCR, was controlled. During the follow-up, his ALT turned to normal on Day 132.

Of all the nine cohabitants, 2 were male and 7 were female. The median age was 35 years, ranging from 28 to 52. Eight of the nine cohabitants were diagnosed with mild COVID-19, while Patient C’s mother as the only exception was never detected positive in RT-qPCR and did not have any respiratory symptoms. Eight of the nine cohabitants were injected with at least two doses of SARS-CoV-2 Vaccine (Vero Cell), Inactivated. The onset dates of 5 (55.6%) cohabitants were earlier than the respective patient. The symptoms were similar in one household. The most common symptom was fever (n = 5, 55.6%), lasting for mostly 2 days (n = 3, 60%). Others included sore throat (n = 2), dry cough (n = 2), fatigue (n = 1), and myalgia (n = 1). The positive result duration and the disease course of the cohabitants were shorter than those of the patients. A significant difference was found in the length of disease course between patients and their cohabitants (*P* < 0.05). Most cohabitants did not accept treatment and none of them received post-negative positive results for RT-qPCR (Table 4).

**Table 4:** The COVID-19 disease course of the patients’ cohabitants

	Relationship	Age (y)	Injected doses of SARS-CoV-2 vaccine	COVID-19 severance†	Onset date compared with the patient (day)‡	Presentation	RT-PCR positive duration (days)	Duration compared with the patient (day) §	Treatment	Relapse
Patient A	mother	32	2	Mild	−7	fever	10	−30	None	None
	Father	36	2	Mild	0	fever	10	−30	None	None
Patient B	Grandmother	52	3	Mild	−2	fever for 2 days	20	−9	None	None
	Mother	28	2	Mild	−1	fever for 2 days	19	−10	None	None
Patient C	Mother	38	1	Uninfected	-	-	0	-	Antipyretic	None
Patient D	Mother	35	2	Mild	−3	fever, myalgia, sore throat for 2 days	14	−5	None	None
	Aunt	49	2	Mild	0	sore throat for 1 day	21	2	None	None
Patient E	Father	28	3	Mild	1	dry cough for > 11 days	5	−13	None	None
	Mother	30	0	Mild	-3	dry cough, fatigue for > 13 days	3	−15	None	None

†The uninfected is defined as cases with no positive results in RT-qPCR. The mild is defined as cases with upper respiratory tract symptoms and normal respiratory system examination. The moderate is defined as cases with abnormal radiological findings as pneumonia without the symptoms of dyspnea and hypoxemia

‡Onset date compared with the patient is defined as the onset date of the co-habitant minus that of the patient. A negative number means the infection of the co-habitant starts earlier than the patient, while 0 means the infection starts on the same day

§Duration compared with the patient is defined as the infection duration of the co-habitant minus that of the patient. A negative number means the infection duration of the co-habitant is shorter than that of the patient, while a positive number means the infection duration is longer than that of the patient

*SARS-CoV-2* Severe Acute Respiratory Syndrome Coronavirus 2, *COVID-19* Corona Virus Disease 2019, *RT-qPCR* real-time reverse transcriptase polymerase chain reaction assay

## Discussion

This is the first cohort of post-LT recipients infected with variant BA.2 (Omicron). The mutations of Omicron variants in the spike protein and the receptor-binding domain affect transmission, disease presentation, and natural or vaccine-induced immunity [34]. Laboratory studies have shown that omicron replicates more in upper airway cells and less in the lungs than Delta [35, 36]. Thus, patients with omicron infection may be more likely to present with fever and upper respiratory symptoms. In developed districts, patients infected with the Omicron variant were younger, with lower protection rate by vaccination, and presented lower rate of dyspnea [2, 37, 38]. The disease severity was lower than that in previous waves [39]. Surprisingly, Omicron variants caused more death and heavier medical burden than previous waves due to its quick spread regardless of vaccination status [3, 8, 37, 40–42]. In our study, fever, instead of upper respiratory symptoms, was the most common symptom in post-LT immunosuppressive children and their cohabitants, supporting the previous studies.

In pediatric population, SARS-CoV-2 seropositivity increased during Omicron variant period compared with Delta waves, implicating either increased transmissibility or reinfection rates [43]. For children with native livers, the severance of the infection was usually milder and there was no significant difference in the risk of hospitalization for young children between Omicron and Delta [44]. The symptoms were generally milder, simpler than those in infected adults and there was usually no demand for intense care unit or mechanical ventilation [37, 38, 44]. For those below 9 years old, the risk of death resulting from disease severe is very low [40]. Children are less threatened by acute or post-acute COVID-19 sequelae [45]. Immunogenicity to SARS-CoV-2 is low in post-LT patients [46]. In other immunosuppressive populations, such as people with advanced HIV, a prolonged SARS-CoV-2 infection may persist for months and silence COVID-19 symptoms for most of its course [41, 47]. Our study found that the length of the infection course was longer than the cohabiting adults, indicating a less effective viral clearance. Yet, the onset of symptoms appeared a few days earlier than RT-qPCR positiveness, accompanied with a low increase in lymphocyte count and CRP in post-LT immunosuppressive children. COVID-19 sequelae did not occur, even in the two relapse children [48]. The low disease severity and prolonged infection during immunosuppression may result from the low inflammatory response against the virus but do not lift the risk of COVID-19 sequelae in this immunosuppressed cohort.

Emphasis has been put on the prevention of COVID infection in post-LT immunosuppressive pediatric patients. A booster vaccine or previous SARS-CoV-2 infection is less effective to activate antibody responses of innate immunity or to achieve an ideal neutralizing activity in Omicron variants than other variants [49–51], but it can still reduce the infection duration and disease severity regardless of the vaccine type [40, 52]. In immunosuppressive solid organ transplant recipients, vaccine-induced Immunoglobin G dividing Immunoglobin A (IgG/IgA) antibody titers against SARS-CoV-2 and the linear B-cell epitopes were reduced, indicating reduced B-cell diversity [49]. Besides, households with one or more children faced larger risks of infection than those without children [53]. The household secondary attack rate of SARS-CoV-2 was higher in the Omicron-dominated waves [54]. In our study, none of the patients accepted the vaccine, while eight of nine cohabitants accepted full doses of inactivated vaccine, and two of them accepted a booster shot, implying that the protection was limited provided by the cohabitants’ vaccination.

There were still some limitations in our study. The number of participants was limited. A prospective, controlled study with a larger number of participants may provide more solid evidence. Besides, it was in the median follow-up time of 203 days after being infected that patients’ infection, graft condition and overall health were controlled at a favorable level. The effect of COVID-19 on long-term graft survival needs further investigation through a longer follow-up.

In conclusion, we presented in detail the characteristics and outcomes of post-LT children infected with SARS-CoV-II and their cohabitants during the COVID-19 outbreak in Shanghai, China, 2022. The infection of all our post-LT patients was associated with in-family transmission. Patients can gain a favorable outcome and prognosis by extenuating or pausing immunosuppressants during and a short time after the SARS-CoV-II infection. These patients were successfully followed up at outpatient department after discharge and did not develop COVID-19 sequelae or reinfection till the last follow-up.

## Data Availability

The datasets used and/or analysed during the current study are available from the corresponding author on reasonable request.

## Abbreviations

ALT: Alanine transaminase
ALP: Alkaline phosphatase
AST: Aspartate transaminase
COVID‐19: Corona Virus Disease 2019
CRP: C‐reactive protein
CsA: Cyclosporin A
CT: Computed Tomography
Ct: Cycle threshold
DB: Direct bilirubin
EBV: Epstein–Barr Virus
GRWR: Graft volume/recipient body weight ratio
HBV: Hepatitis B Virus
Ig: Immunoglobin
IVIG: Intravenous immunoglobulin
LDLT: Living donor liver transplantation
LT: Liver transplant
MMF: Mycophenolate mofetil
PA: Prednisone acetate
PCR: Polymerase Chain Reaction
PTLD: Post‐transplant lymphoproliferative disease
q12h: Every 12 h
qd: Every day
RT-qPCR: Real-time reverse transcriptase polymerase chain reaction assay
SARS‐CoV‐2: Severe Acute Respiratory Syndrome Coronavirus 2
SLT: Split liver transplantation
TAC: Tacrolimus
TB: Mycobacterium tuberculosis
WBC: White blood cell
WLT: Whole liver transplantation
γ-GT: Gamma‐glutamyl transferase
